# Retraction: Gradual Rarefaction of Hematopoietic Precursors and Atrophy in a Depleted microRNA 29a, b and c Environment

**DOI:** 10.1371/journal.pone.0187454

**Published:** 2017-10-30

**Authors:** The PLOS ONE Editors

The *PLOS ONE* Editors retract this publication, as we have been advised that the authors did not have appropriate rights or permissions to publish the data.

After publication, concerns were raised about the ownership of data reported in this article. This matter was reviewed by the Office of Research Compliance at Ohio State University, where the research took place. The Office of Research Compliance advised that the data were generated in laboratories at Ohio State University and were published without permission of the principal investigators, in breach of the University’s Research Data Policy.

In the light of the recommendation of the Ohio State University, the *PLOS ONE* Editors retract this publication.

The corresponding author, Stefan Costinean, does not agree to this retraction.
